# Cryopreservation of Whole Tumor Biopsies from Rectal Cancer Patients Enable Phenotypic and In Vitro Functional Evaluation of Tumor-Infiltrating T Cells

**DOI:** 10.3390/cancers13102428

**Published:** 2021-05-17

**Authors:** Frank Liang, Azar Rezapour, Peter Falk, Eva Angenete, Ulf Yrlid

**Affiliations:** 1 Department of Microbiology and Immunology, Institute of Biomedicine, Sahlgrenska Academy, University of Gothenburg, 405 30 Gothenburg, Sweden; frank.liang@gu.se (F.L.); azar.rezapour@gu.se (A.R.); 2 Department of Surgery, Fibrinolysis Laboratory, Institute of Clinical Sciences, Sahlgrenska Academy, University of Gothenburg, 416 85 Gothenburg, Sweden; peter.falk@surgery.gu.se; 3 Department of Surgery, Sahlgrenska University Hospital/Östra, Region Västra Götaland, 413 45 Gothenburg, Sweden; 4 Department of Surgery, SSORG—Scandinavian Surgical Outcomes Research Group, Institute of Clinical Sciences, Sahlgrenska Academy, University of Gothenburg, 416 85 Gothenburg, Sweden

**Keywords:** rectal cancer, tumor-infiltrating lymphocytes, γδ T cells, MAIT cells, IFN-γ responses, flow cytometry

## Abstract

**Simple Summary:**

Colorectal cancer (CRC) remains the third most common malignancy. Tumor-infiltrating lymphocytes (TILs) have emerged as correlates to CRC patient outcome after treatment. The pro- or anti-tumor responses of TILs are usually assessed in cell suspensions of fresh tumors that were surgically removed a few hours earlier. We propose a platform for concurrent enumeration and in vitro functional evaluation of TILs in cryopreserved tumor biopsies, offering the benefit of postponing tumor processing and analyses of TILs in cell suspensions until clinical post-treatment responses are established. Our platform is practical considering the inconsistent time when patient samples become available for research purposes and can be readily utilized by other laboratories. With a fresh portion of tumor biopsies as benchmark, we validated the recovery of viable TILs capable of interferon (IFN)-γ responses in the cryopreserved portion of same biopsies. Ultimately, this platform could provide sufficient information on TILs, to also predict patient outcome after CRC treatments.

**Abstract:**

TILs comprise functionally distinct conventional and unconventional T cell subsets and their role in responses to CRC treatments is poorly understood. We explored recovery of viable TILs from cryopreserved tumor biopsies of (chemo)-radiated patients with rectal cancer to establish a platform for retrospective TIL analyses of frozen tumors from pre-selected study cohorts. Frequencies of TIL subsets and their capacity to mount IFN-γ responses in cell suspensions of fresh vs. cryopreserved portions of the same tumor biopsies were determined for platform validation. The percentages and proportions of CD4+ TILs and CD8+ cytotoxic T lymphocytes (CTLs) among total TILs were not affected by cryopreservation. While recovery of unconventional γδ T cells and mucosal-associated invariant T cells (MAIT cells) was stable after cryopreservation, the regulatory T cells (Tregs) were reduced, but in sufficient yields for quantification. IFN-γ production by in vitro-stimulated CD4+ TILs, CTLs, γδ T cells, and MAIT cells were proportionally similar in fresh and cryopreserved tumor portions, albeit the latter displayed lower levels. Thus, the proposed platform intended for TIL analyses on cryopreserved tumor biobank biopsies holds promises for studies linking the quantity and quality of TIL subsets with specific clinical outcome after CRC treatment.

## 1. Introduction

CRC is the third most common malignancy and the second most common cause of cancer-related mortality [1,2]. It has been shown that substantial numbers of intratumoral T cells, referred to as TILs, are associated with specific prognosis [3,4,5,6,7]. Recently, the in situ level of TILs plus their location and activation status have emerged as predictive correlates to clinical responses to treatment modalities in CRC, including immunotherapy [8,9] and chemotherapy alone [10,11] or in combination with radiotherapy [12,13]. The TILs in these studies were identified histologically by cellular morphology or staining of a few T cell markers. Histology on archival tumors has the benefit of discerning intratumoral location of TILs [3,4,5,6,7,14], but has limitations in simultaneous analyses of the extensive number of immunological markers necessary for the identification of TIL subsets.

TILs consist of conventional and unconventional T cell subsets. Among conventional TILs defined by their expression of αβ T cell receptors (TcRs), CD8+ CTLs with tumor-killing capacity are considered the main effector cells in tumor control and/or elimination. In contrast, the CD4+ TILs comprise several T cell subsets with different roles in tumor immunity, including the immunosuppressive Tregs [4,5,6]. The unconventional γδ T cells and MAIT cells, which were initially recognized for their involvement in anti-microbial responses, have recently gained interest in CRC treatment strategies [15,16]. Of note, the γδ T cells in CRC have shown to correlate with longer disease-free survival [17] or support the pro-tumor myeloid-derived suppressor cells [18]. Increased levels of MAIT cells were reported to associate with poor prognosis [19], despite reports on their proficient production of molecules associated with tumor-killing [20,21]. In general, functional anti-tumor responses by TILs are represented by the production of effector cytokine IFN-γ. These IFN-γ responses stem from either the combined efforts of all TILs, or more likely, from specific TIL subsets. Thus, studies linking specific clinical post-treatment outcome with the composition and functional capacity of TIL subsets requires cell suspensions analyzed by flow or mass cytometry.

Here, we propose and validated a platform that enables postponing of phenotypic and in vitro functional evaluation of TIL subsets in cryopreserved CRC biopsies to any preferred time. For validation, epithelial and lamina propria cell suspensions obtained from fresh and cryopreserved portions of the same tumor biopsies were compared with regards to the frequency and functional capacity of TIL subsets. We show that tumor cryopreservation is a prerequisite for recovery of viable TILs for subset quantification simultaneous with determination of their inducible IFN-γ responses. The proposed platform would enable extensive TIL subset analyses when randomly selected tumor biobank biopsies are forwarded to blinded investigators for unbiased evaluation. Further, delayed access to research samples due to extended time for completion of surgery makes tumor processing in direct conjunction with sample availability inconvenient. Therefore, the proposed platform also encompasses practical and logistical advantages.

## 2. Results

### 2.1. The Cryopreservation Method Is Key for Flow Cytometric Analyses of Tumor Suspensions

**Figure 1:**
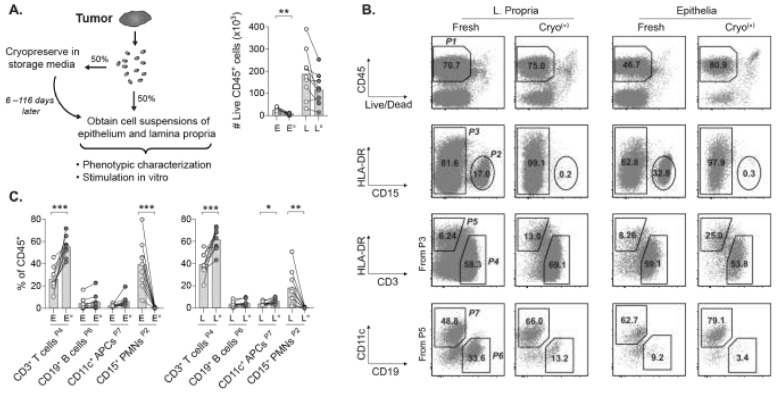
Recovery of CD45+ leukocytes in cryopreserved tumors of rectal cancer patients. (**A**) Experimental overview and number of viable CD45+ leukocytes. (**B**) Gating strategy for epithelial and lamina propria cell suspensions of fresh and cryopreserved portions of the same tumor. The CD45+ leukocytes (*P1*) comprise CD15+ PMNs (*P2*), and the remaining leukocytes (*P3*) are divided into CD3+ HLA-DR dim/– T cells (*P4*) and HLA-DR+ APCs (*P5*). The CD19+ B cells (*P6*) and CD11c+ myeloid APCs (*P7*) are from *P5*. Percentages of parent population are shown. (**C**) Frequencies of cell subsets within CD45+ leukocytes. E and L denotes epithelia and lamina propria, respectively. ° indicates the cryopreserved tumor portion. Bars show the mean. (* *p* < 0.05, ** *p* < 0.01, *** *p* < 0.001, Welch’s *t* test).

Isolation of leukocytes from cryopreserved tissue is generally perceived as futile since low yields of viable cells are typically expected. Whether this applies to whole tumor biopsies that are frozen in conventional cryopreservation media (i.e., storage media) for peripheral blood mononuclear cells (PBMCs) remains undetermined. In our attempts to recover TILs from cryopreserved tumors, fresh rectal tumor biopsies were first cut into small pieces and then randomly divided into two equal portions (Figure 1A). One of the portions was immediately separated into fractions of tumor epithelium and lamina propria, processed into cell suspensions, and used as benchmark for platform validation. The other portion was cryopreserved in storage media for 6–116 days, then thawed and processed the same way as the benchmark. A quarter of the epithelial and lamina propria cell suspensions of fresh or cryopreserved tumor portions were stained for flow cytometry with fluorescently conjugated antibodies (Supplementary Table S1) and the remaining was used for in vitro-stimulation of TILs. Enumeration of viable CD45+ leukocytes in cell suspensions from tumor epithelia and lamina propria indicated significant loss of leukocyte numbers mainly in the epithelium after cryopreservation (Figure 1A). Although the lamina propria from a few cryopreserved tumors displayed reduced numbers of leukocytes, this was not statistically significant. Nevertheless, viability of intratumoral leukocytes was completely abolished when tumors were cryopreserved without storage media, or with other commonly used tissue preservation strategies such as OCT and RNAlater (Supplementary Figure S1). Thus, specific cryopreservation conditions have to be met for the recovery of sufficient leukocytes for flow cytometric analyses of frozen tumor biopsies of limited size.

### 2.2. Viable TILs Can Be Recovered from Cryopreserved Tumors for Multiparametric Phenotyping

Despite the reduction in leukocyte numbers in cryopreserved tumor fractions, multiple immune cell subsets were readily identified from the viable CD45+ leukocyte population (Figure 1B). Of note, there was a substantial loss of CD45+ CD15+ polymorphonuclear (PMN) cells in both the epithelial and lamina propria fractions of cryopreserved tumors (Figure 1B,C and Supplementary Table S2). No significant reduction in numbers was observed for other leukocyte subsets (Supplementary Table S2). This suggests loss of PMNs as the main reason for decreased numbers of CD45+ leukocytes. The fragility of PMNs found here is consistent with a previous report where tumor suspensions, rather than whole tumor biopsies, were cryopreserved [22]. As a consequence of decreased PMNs, the frequency of CD3+ TILs among CD45+ leukocytes was significantly increased. Overall, CD19+ B cells and HLA-DR+ CD11c+ antigen presenting cells (APCs) were slightly increased after cryopreservation (Figure 1C). Of note, TIL frequencies in separate aliquots of cryopreserved portions of the same tumor were similar when these aliquots were thawed, processed, and analyzed at different occasions, which indicate robust recovery of viable TILs (Supplementary Figure S2). Furthermore, similar trends in leukocyte frequencies were observed in fresh vs. cryopreserved macroscopically tumor-free mucosa adjacent to the tumor (Supplementary Figure S3).) This suggests the feasibility of applying the proposed platform also to tumor-free control tissues.

### 2.3. Frequencies and Proportions of Major TIL Subsets Remain Stable in Cryopreserved Tumors

**Figure 2:**
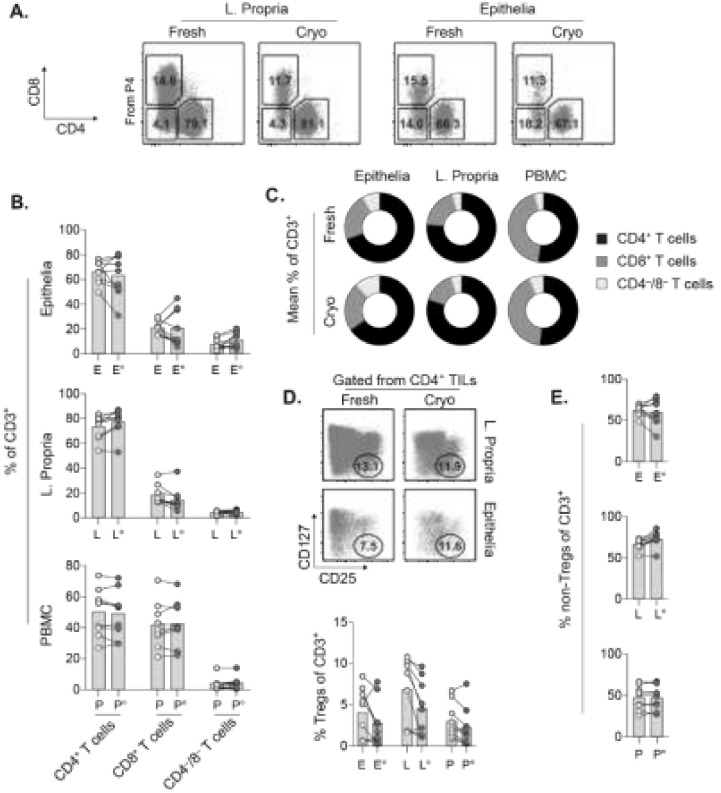
Determination of T cell subset frequencies within total CD3+ T cells. (**A**) Gating of T cell subsets from total CD3+ T cells (*P4* in Figure 1B), which contain the CD4+ TILs, CD8+ CTLs, and CD4– CD8– population. Percentages of parent population shown. (**B**) Compiled data on T cell subset frequencies within total CD3+ T cells. (**C**) Proportions of CD4+ TILs, CD8+ CTLs, and CD4– CD8– TILs in tumor compartments and their counterparts in PBMCs. (**D**) Gating of CD4+CD25+CD127dim/–Tregs and their frequency among total TILs or T cells in PBMCs. Percentages of parent population shown. (**E**) Frequency of non-Tregs (i.e., CD4+ T cells outside the Treg gate) in total TILs or circulating PBMCs. E, L, and P denotes epithelia, lamina propria, and PBMC, respectively. ° indicates cryopreserved samples. Bars show the mean. Welch’s *t* test.

As subsets of TILs have distinct roles in tumor immunity [3,4,5,6,7,8], we evaluated whether cryopreservation impacts the frequencies of CD4+ TILs, CD8+ CTLs, and CD4– CD8– TILs within total CD3+ TILs (Figure 2A). We observed stable recovery of TIL subsets in comparison of fresh vs. cryopreserved tumors (Figure 2B). Since PBMCs are relatively accessible research samples, we also compared recovery of TILs to the stability of circulating T cell frequencies in fresh and frozen PBMCs from the same patients. The robustness in the recovery of major TIL subsets was close to that of PBMCs, which unlike the tumors, were not isolated by enzymatic digestion. This suggests that processing of fresh or cryopreserved tumors had a minimal effect on the integrity of cell surface markers, by which the TIL subsets were defined. In line with the T cell subsets in PBMCs, the proportional hierarchy of TIL subsets in cell suspensions from fresh vs. cryopreserved tumors also showed a high degree of similarity (Figure 2C). Thus, minor differences in TILs subset frequencies observed in individual fresh and cryopreserved tumors were negligible when proportional distribution was assessed.

### 2.4. Tregs Are Reduced in Cryopreserved Tumors but Sufficient for Phenotypic Analyses

Next, we advanced our subset analyses to also include the CD4+CD25+CD127dim/–Tregs (Figure 2D). These are mainly suppressive T cells and are frequently enriched in solid tumors [23,24]. The vast majority of Tregs in fresh and cryopreserved tumors expressed the transcription factor forkhead box P3 (Foxp3) (Supplementary Figure S4), as previously reported for CD4+CD25+CD127dim/–Tregs [25,26,27]. Although recovery of CD4+ TILs was stable after cryopreservation, the frequency of Tregs in total TILs was consistently reduced mainly in the lamina propria (Figure 2D). However, the overall decrease in Treg frequency (Figure 2D) or numbers (Supplementary Table S2) after cryopreservation was not statistically significant. To rule out whether reduction of Tregs in cryopreserved tumors was a consequence of their altered expression of CD25 and CD127, we assessed the CD4+ TILs outside the Treg gate (referred to as non-Tregs). We did not observe significant changes in the frequency of non-Tregs within total CD3+ TILs, which would be expected following downregulation of CD25 and/or upregulation of CD127. Altogether, cryopreserved tumors yielded lower levels of Tregs, but they were in sufficient numbers for phenotypic analyses. Studies that require highly purified Tregs would therefore require several cryopreserved biopsies from the same tumor for adequate yield.

### 2.5. Concurrent Assessment of Unconventional T Cell Subsets in Cryopreserved Tumors

**Figure 3:**
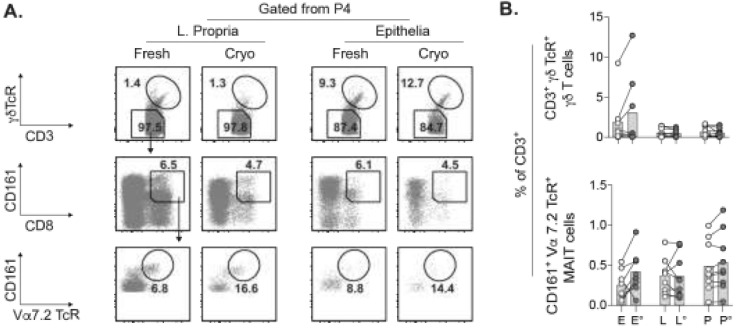
Quantification of unconventional γδ T cells and MAIT cells. (**A**) γδT cells expressing γδ TcR from total CD3+ TILs (*P4* in Figure 1B). CD161+ Vα7.2 TcR+ MAIT cells are from the CD8+ TILs contained in the CD3+ γδ TcR– population. Percentages of parent population shown. (**B**) Frequencies of intratumoral γδ T cells and MAIT cells and their counterparts in PBMCs within total T cells. E, L, and P denotes epithelia, lamina propria, and PBMC, respectively. ° indicates cryopreserved samples. Bars show the mean. Welch’s *t* test.

Our characterization of small TIL subsets such as the Tregs were subsequently extended to include the unconventional T cells. These T cells are comprised of γδ T cells expressing the γδ TcR and CD8+CD161+Vα7.2 TcR+ MAIT cells, which have been described to promote or counteract anti-tumor immunity [17,19,28]. Both γδ T cells and MAIT cells were readily detected in cell suspensions of fresh and cryopreserved tumors (Figure 3A). Whereas the frequency of γδ T cells among total CD3+ TILs was similar in fresh vs. cryopreserved PBMCs and tumor lamina propria, some cryopreserved tumors had increased γδ T cell levels in the epithelia (Figure 3B). MAIT cells were recovered after cryopreservation with the same trend as for the γδ T cells with regards to frequency and numbers (Figure 3B and Supplementary Table S2). There were no significant differences in γδ T cell or MAIT cell frequency or numbers between fresh vs. cryopreserved tumors.

### 2.6. TILs Retain Inducible IFN-γ Responses after Cryopreservation

**Figure 4:**
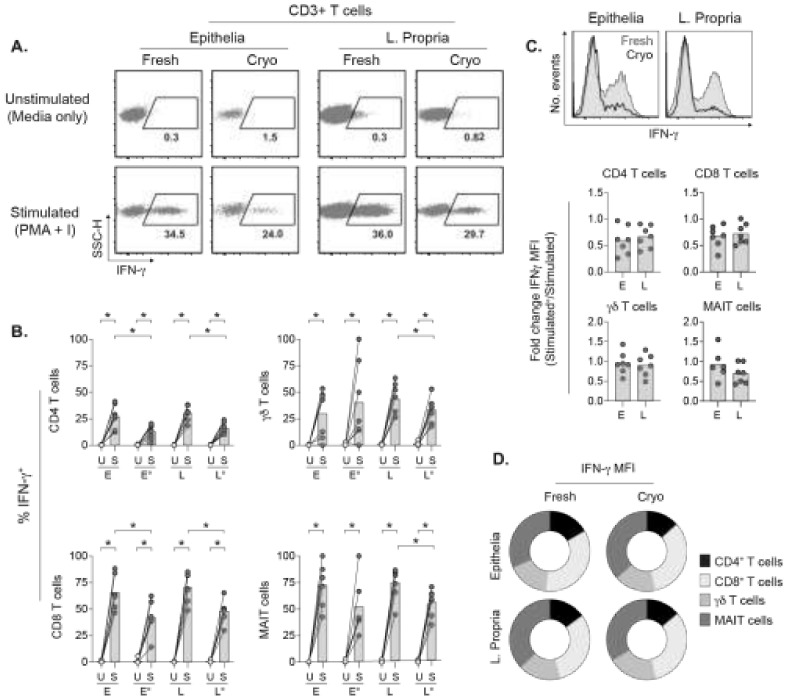
IFN-γ responses are maintained in TILs after cryopreservation. (**A**) IFN-γ expressed by CD3+ TILs stimulated with PMA and ionomycin or left unstimulated. Percentages of parent population shown. (**B**) Frequencies of IFN-γ+ TIL subsets in unstimulated (U) or stimulated (S) suspensions. (**C**) Histograms of IFN-γ staining on CD3+ TILs in fresh and cryopreserved tumors with IFN-γ staining intensity on x-axis and cell event numbers on y-axis. Fold changes represent mean fluorescence intensity (MFI) of IFN-γ staining for the indicated TIL subsets in frozen tumor portion, divided by MFI of the same TIL subset in the fresh portion. (**D**) Proportional distribution of IFN-γ+ TIL subsets. E and L denotes epithelia and lamina propria, respectively, and ° indicates cryopreserved samples. Bars show the mean. (* *p* < 0.05, Wilcoxon signed-rank test).

Activated T cells secrete large amounts of IFN-γ that regulates different aspects of innate and adaptive immunity in tumor control and/or elimination [17,20,29]. To evaluate the capacity of TILs to mount IFN-γ responses after cryopreservation, cell suspensions were stimulated overnight with low concentrations of phorbol 12-myristate 13-acetate (PMA) and ionomycin. Compared to unstimulated controls, total CD3+ TILs from fresh or cryopreserved tumors readily produce IFN-γ upon polyclonal stimulation (Figure 4A). Unconventional γδ T cells and MAIT cells were excluded from the intratumoral αβ T cells (i.e., αβ TILs) to enable separate analysis of conventional and unconventional TILs. Relative to fresh tumor portion, frequencies of IFN-γ+ TIL subsets were significantly decreased after cryopreservation. Among the αβ TILs in fresh or cryopreserved tumors, IFN-γ production by CD8+ TILs exceeded that of CD4+ TILs in epithelia and lamina propria (Figure 4B). Although scarce in numbers, both γδ T cells and MAIT cells with inducible IFN-γ responses were detected in cryopreserved tumors. To compare the efficiency of IFN-γ production, we evaluated the intensity of IFN-γ signals expressed as mean fluorescence intensity (MFI) (Figure 4C). The histograms representing IFN-γ expressed by the total CD3+ TILs and the MFI fold change indicated that CD4+ or CD8+ αβ TILs in cryopreserved tumors expressed lower IFN-γ levels. Among unconventional T cells, the γδ T cells and MAIT cells produced varying amounts of IFN-γ depending on the specific patient. Importantly, the proportional hierarchy of TIL subsets based on IFN-γ MFI of all stimulated cell suspensions was similar overall (Figure 4D).

In summary, we proposed an analysis platform for cryopreserved tumor biobank biopsies that can be readily utilized by other investigators with relatively little effort. This platform was validated by comparisons of fresh vs. cryopreserved portions of the same rectal tumor biopsies with regards to subset phenotype, ex vivo frequency, and in vitro functional capacity of TILs. We showed the sturdy recovery of CD4+ TILs, CD8+ CTLs, γδ T cells, and MAIT cells after cryopreservation. The relatively scarce γδ T cells, MAIT cells, as well as the reduced Tregs were quantified in cryopreserved tumors. In addition, we observed lower frequencies of IFN-γ+ in vitro-stimulated TIL subsets in frozen tumors, although the IFN-γ expression levels in fresh and frozen tumor fractions were proportionally similar.

## 3. Discussion

Isolation of viable TILs from cryopreserved whole tumors is considered an insurmountable challenge and have been left unexplored. However, tumor biopsies are normally not frozen and stored in media optimized for e.g., PBMCs, which prompted us to explore TIL recovery after cryopreservation. We utilized several tissue preservation protocols in our attempts to optimize TIL recovery in frozen tumor biopsies from a small cohort of treated patients undergoing rectal cancer surgery. We found substantial cell death with all protocols except cryopreservation in storage media, which are frequently used commodities in laboratories maintaining cell cultures. These data clearly show that specific cryopreservation condition is required to characterize multiple TIL subsets in tumor cell suspensions. To address the compartmentalization of intratumoral leukocytes, we generated suspensions of epithelia and lamina propria cells separately.

Beyond the TILs, suspensions from relatively small amounts of fresh and cryopreserved pieces of the same tumors revealed substantial loss of CD15+ PMNs, which are primarily neutrophils. In contrast, the recovery of CD11c+ APCs, consisting of macrophages and dendritic cells, from cryopreserved tumors was fairly stable. Neutrophils are relatively short-lived, which may explain their tendency to perish after cryopreservation. Hence, flow cytometric analyses of intratumoral PMNs require fresh clinical material. Whether cryopreservation methods beyond those that we have tested, spare PMNs remains to be determined.

The loss of PMNs distorts the relative TIL frequency within CD45+ leukocytes in comparisons between fresh vs. cryopreserved tumors. Thus, the robustness in recovery of major TIL subsets (CD4+ TILs, CD8+ CTLs, and CD3+ CD4– CD8– TILs) was more accurately represented by their frequency and proportions among total CD3+ TILs. Although the recovery of CD4+ TILs remains stable in thawed tumors, Tregs within total TILs were reduced, and most consistently in the lamina propria fraction. The stability of circulating Tregs in fresh vs. cryopreserved PBMCs show that Tregs were not affected by cryopreservation itself. Intratumoral Tregs have phenotypically been described as activated and exhausted by the tumor microenvironment [23,30]. This could theoretically render them particularly sensitive to tumor processing after cryopreservation. Whether such potential sensitivity manifests in downregulation of the Treg markers used is unlikely, as there was no overall increase of non-Tregs within CD4+ TILs. Nonetheless, the small amount of cryopreserved tumor tissue used for each experiment enabled efficient enumeration of Tregs by surface markers or intranuclear Foxp3 expression.

Detection of relatively small TIL populations such as the Tregs in cryopreserved tumors led us to also explore the unconventional T cells, which are usually also scarce in tumors. We readily identified both γδ T cells and MAIT cells, which were increased in some of the cryopreserved tumors. Due to the rarity of unconventional TILs, it is plausible that their distribution within the portions of fresh and cryopreserved tumor is uneven, which could give rise to variation in their frequencies. Thus, analyses of γδ T cells and MAIT cells may require suspensions derived from the entire tumor biopsy rather than a portion of the same biopsy.

Aside from the magnitude of T cell subset homing to tumors, their functional capacity provides an additional and vital feature of intratumoral T cell responses. TILs have the potential to secrete multiple cytokines that regulate different aspects of tumor immunity [4,17,20,24]. Given the importance of IFN-γ in intratumoral responses [31], we focused on this cytokine. Indeed, IFN-γ was inducible by PMA and ionomycin stimulation in all TIL subsets examined, and spontaneous production by unstimulated TILs, whether fresh or thawed, was not detected. Frequency of IFN-γ+ TILs and expression of IFN-γ were generally lower in stimulated TILs of cryopreserved tumors. In contrast, a previous report comparing fresh vs. thawed cell suspensions of rectal tumors showed higher percentages of IFN-γ+ CTLs in the frozen suspensions [22]. However, the thawed suspensions in that study were rested overnight prior to stimulation and whether other TIL subsets responded in similar manner was not addressed. Our approach of not resting the cells prior to stimulation avoided potential alteration of cell surface markers essential for identification of TIL subsets, particularly those in scant numbers, which could lead to misrepresentation of IFN-γ+ TIL subset frequencies. However, despite lower IFN-γ production, the proportional hierarchy for the levels of IFN-γ among αβ T cell subsets, γδ T cells and MAIT cells remained unperturbed in the fresh vs. cryopreserved tumor groups. Importantly, evaluation of inducible functionality of TIL subsets, such as cytokine secretion, can at the present only be assessed with cell suspensions.

Our proposed platform provides the significant advantage of initiating quantification and functional in vitro analyses of TILs before or after clinical responses have been confirmed in treated patients. To avoid potential bias caused by sample selection, cryopreserved tumors from patients that responded beneficially to specific treatments or not can be randomly dispensed to blinded investigators for TIL analyses in one single occasion and thereby limit inter-experimental differences. Aside from the practical benefit of processing frozen tumors at any chosen time, performing initial TIL analyses on a limited number of randomly selected tumor biopsies also enables efficient use of costly reagents and clinical samples. Other advantages offered by our platform includes analyses of inducible and/or functionally regulated gene and protein expression on specific TILs in non-fixed tumors for immune checkpoint inhibition therapy. Previous reports on establishment of patient-derived xenograft models with various frozen tumors [32,33] also suggest additional potential for our platform. Integration of data from these abovementioned assays may facilitate design of personalized therapy. Finally, access to multiple aliquots of the same cryopreserved tumor also allows revisiting samples from the same patients to address additional questions that arise including, for example, previously unexplored TIL subsets and/or novel markers targetable by immunotherapy.

## 4. Materials and Methods

### 4.1. Rectal Cancer Patients

The study was approved by the Regional Board of Ethics in Gothenburg, Sweden and signed informed consent were received from included patients with rectal cancer. Surgery was performed after concluded radiotherapy alone (5 × 5 Gy) or radiotherapy (5 × 5 Gy) followed by chemotherapy (4 cycles of capecitabine and oxaliplatin). Median time between last treatment and surgery was 4 days (Min–Max: 1–143 days).

### 4.2. Cryopreservation of Tumors

The surgically resected tumors were immediately forwarded to research nurses in the surgical department for cleaning of tumor tissue by removal of macroscopically visible adipose, muscle, and connective tissues as previously described [34]. After cleaning, the tumors were cut into 3 × 3 mm pieces with a scalpel and randomly divided into two portions (5 pieces per portion). One of these portions was submerged in 1 mL storage media consisting of 90% fetal bovine serum (FBS) (Gibco, Thermo Fisher, Carlsbad, CA, USA) and 10% Dimethyl sulfoxide (DMSO) (Fisher Scientific, Thermo Fischer) for cryopreservation in liquid nitrogen, until use (6–116 days later). The other portion (i.e., the fresh tumor portion) was submerged in complete media consisting of RPMI 1640 supplemented with 10% fetal bovine serum, 1% penicillin/streptomycin, 1% Hepes buffer, and 0.1% Gentamycin (Gibco) prior to transport on ice and processed into cell suspensions within 1hr. For comparisons between separate cryopreserved tumor aliquots from the same patients, both portions were frozen in storage media. For some patients, macroscopically tumor-free rectal mucosa from the resectate that was approximately 2–5 cm away from the tumor border was collected in parallel. These mucosal tissue were cleaned, cryopreserved, and processed into cell suspension using the same protocols as for tumors. To evaluate storage with other commercially available reagents, tumor pieces were cryopreserved either without storage media, or in RNAlater (Invitrogen, Waltham, MA, USA) or OCT cryomount (Histolabs, Gothenburg, Sweden).

### 4.3. PBMC Isolation

Venous blood was collected into EDTA vacutainers (BD, San Jose, CA, USA) during surgery. PBMCs were isolated by Ficoll gradient (GE Healthcare, Uppsala, Sweden) using standard protocol. Briefly, blood was diluted 1:2 with PBS and layered on top of room-temperatured Ficoll gradient in 50 mL-conical tube (Sarstedt). To obtain interphase layer containing the PBMCs, overlayed blood was centrifuged for 20 min at 870× *g* without break and with the lowest acceleration. PBMCs were collected from the interphase and washed twice with phosphate-buffered saline (PBS) at 500× *g* for 5 min at room temperature with full acceleration and a break. The isolated PBMCs were used directly or cryopreserved in storage media.

### 4.4. Generation of Single Cell Suspensions from Fresh and Cryopreserved Tissues

The 3 × 3 mm tumor pieces were transferred to a flat-bottomed 40 mL-tube (Sarstedt, Nümbrecht, Germany), placed on magnetic stirrer set on moderate rotation, and washed four times at 37 °C (15 min/wash) with 15 mL of Hanks’ balanced salt solution without Mg^2+^ or Ca^2+^ (HBSS) (Gibco) containing 2% FBS (Gibco), 1% Hepes buffer, and 2 mM EDTA (Lonza, Basel, Switzerland) in each wash. These washes were aspirated with disposable Pasteur pipettes (Sarstedt) and collected into a 50 mL-conical tube (Sarstedt) to obtain the tumor epithelial fraction, while the remaining tissue in the flat-bottomed tube represented the lamina propria fraction. When all four washes were collected, the epithelial fraction was pelleted by centrifugation at 500× *g* for 5 min at room temperature with full acceleration and break. After discarding supernatant from the washes, the pelleted epithelial fraction in the conical tube was transferred to a flat-bottomed 40 mL-tube. Subsequent to an additional wash with 15 mL of supplemented HBSS without EDTA on a magnetic stirrer for 15 min, the supernatants were aspirated and discarded. Both epithelial and lamina propria fractions were then enzymatically digested in their respective 40 mL-tubes on magnetic stirrer with 7 mL of complete media containing Liberase TM (70 µg/mL) (Roche, Basel, Switzerland) and DNase I (20 µg/mL) (Sigma, St. Louis, MO, USA) for 1 h at 37 °C. Enzyme activity of Liberase was quenched with 1 mL FBS and cell suspensions were filtered through 40 µm cell strainer (Thermo Fisher) before washing with PBS at 500× *g* as described above.

### 4.5. Thawing of Cryopreserved Tissues

Cryopreserved portion of tumor or rectal mucosa were thawed in a 37 °C water bath and tissues were immediately transferred to 15 mL-tube (Sarstedt) and added 10 mL PBS. Tissues were washed twice by inverting the tube back and forth 5 times. When tissue pieces were sedimented, supernatant from each wash was aspirated with disposable Pasteur pipette (Sarstedt) and discarded. Washed tissues were then transferred to flat-bottomed 40 mL-tube for the generation of the single cell suspension as described above.

### 4.6. Flow Cytometry

A quarter of the obtained cell suspensions of tumor biopsies and 1.5 million PBMCs in 60 µL PBS were transferred to 5 mL-polystyrene tubes (Corning, Amsterdam, Netherlands) and 40 µL of Zombie Red Fixable viability dye (Biolegend, San Diego, CA, USA) was added and diluted 1000-fold in PBS. This was followed by 20 min incubation with a cocktail of fluorescently conjugated monoclonal antibodies for staining of cell surface markers (Supplementary Table S1). Subsequent to PBS wash at 500× *g* for 5 min, stained cells were fixed with 2% paraformaldehyde in PBS and spiked with AccuCount beads (Spherotech, Lake Forest, IL, USA) for calculation of cell numbers according to the manufacturer’s protocol. For intranuclear Foxp3 staining, eBioscience Foxp3/Transcription Factor Staining Buffer Set (Invitrogen) was used as per manufacturer’s instructions. Briefly, after staining of surface markers, cells were incubated with 1 mL fixation/permeabilization buffer for 30 min and washed with 2 mL of 1× permeabilization buffer for 5 min at 500× *g*. Thereafter, cells were incubated with the fluorescently conjugated anti-Foxp3 antibody (Supplementary Table S1) for 30 min followed by an additional wash with 1× permeabilization buffer. All staining steps were performed at room temperature. A total of 2 × 10^5^–1 × 10^6^ events/sample were acquired using the BD LSRFortessa flow cytometer and analyzed with FlowJo v. 9.9.6 (Tree Star, Ashland, OR, USA).

### 4.7. In Vitro Stimulation

Four million PBMCs and the remaining cell suspensions from fresh and cryopreserved tumors were divided in half and transferred to two 5 mL-polystyrene tubes (Corning). Cells were suspended in 1 mL complete medium with or without 0.01 µg/mL PMA (Sigma) and 0.25 µg/mL ionomycin (Sigma). Cytokine release was inhibited by 10 µg Brefeldin A (Sigma) that was added directly prior to culture at 37 °C and 5% CO_2_ atmosphere. After an overnight culture, cells were washed with PBS prior staining of surface markers and viability as described above. Subsequently, the stained cells were fixed and permeabilized using a Fixation/Permeabilization Solution Kit (BD) according to manufacturer’s protocol. Briefly, cells were incubated with a 0.25 mL fixation/permeabilization solution for 20 min and washed with 1 mL of 1× Perm/Wash buffer at 500× *g* for 5 min. The cells were then stained with anti-IFN-γ antibody (Supplementary Table S1) for 20 min, followed by a final wash with 1× Perm/Wash buffer. All staining steps were performed at room temperature.

### 4.8. Statistical Analysis

Individual data are shown together with the mean (bars). Statistical analyses were performed using Welch’s t test or Wilcoxon signed-rank test supplied by the GraphPad Prism software, v.9.1.0. (San Diego, CA, USA) and considered significant at * *p* < 0.05. Absence of asterisk indicates *p* > 0.05.

## 5. Conclusions

The platform proposed in this study enabled efficient phenotypic and functional characterization of multiple TIL subsets in cryopreserved rectal tumor biopsies of limited size. The practical advantages and possibilities to associate clinical responses to treatment with ex vivo and in vitro TIL responses merits consideration of our TIL analysis strategy as a valid platform for retrospective studies of optimally frozen CRC biobank biopsies. In the long run, associations between specific post-treatment outcome and the composition or functional capacity of TILs may provide sufficient grounds for this platform to instead predict responses to CRC treatments.

## Data Availability

No new data were created or analyzed in this study. Data sharing is not applicable to this article.

## Supplementary Materials

The following are available online at https://www.mdpi.com/article/10.3390/cancers13102428/s1, Figure S1: Assessment of TIL recovery with distinct cryopreservation protocols. Figure S2: Comparison of TIL frequencies in aliquots of the same rectal tumors. Figure S3: Generation of cell suspensions of cryopreserved rectal mucosa. Figure S4: Foxp3 expression by TILs in fresh and cryopreserved rectal tumors. Table S1: Fluorescence-conjugated monoclonal antibodies for flow cytometry. Table S2: Mean ± standard deviation of leukocyte subsets in fresh and cryopreserved rectal tumor suspension.
