# Supplementary material for: Cryopreservation of Whole Tumor Biopsies from Rectal Cancer Patients Enable Phenotypic and In Vitro Functional Evaluation of Tumor-Infiltrating T Cells
Source: Cancers (Basel). 2021 May 17;13(10):2428. doi: 10.3390/cancers13102428 (PMC8155904; doi:10.3390/cancers13102428)
Supplement: Supplementary file 1 [file cancers-13-02428-s001.zip › cancers-1015277-supplementary.pdf]

cancers

MDPI

Supplementary Materials

Cryopreservation of Whole Tumor Biopsies from Rectal Cancer Patients Enable Phenotypic and In Vitro Functional Evaluation of Tumor-Infiltrating T Cells

Frank Liang, Azar Rezapour, Peter Falk, Eva Angenete and Ulf Yrlid

Table S1. Fluorescence-conjugated monoclonal antibodies for flow cytometry.

Antibody	Clone	Manufacturer
CD45	HI30	BD Biosciences
CD3	UCHT1	BD Biosciences
CD8	RPA-T8	BD Biosciences
CD11c	B-ly6	BD Biosciences
CD15	HI98	BD Biosciences
CD19	SJ25C1	BD Biosciences
CD25	2A3	BD Biosciences
HLA-DR	G46-6	BD Biosciences
TCR γδ	B1	BD Biosciences
IFN-γ	B27	BD Biosciences
CD4	OKT4	Biolegend
CD161	HP3G-10	Biolegend
CD127	A019D5	Biolegend
TCR Vα7.2	3C10	Biolegend
FoxP3	150D	Biolegend

Table S2. Mean ± standard deviation of leukocyte subsets in fresh and cryopreserved rectal tumor suspension.

Immune cell subset	E (x 10^3)	E° (x 10^3)	L (x 10^3)	L° (x 10^3)	p[§] E vs. E°	p[§] L vs. L°
$CD45^+$ Leukocytes	25 ± 13	8 ± 4	186 ± 112	115 ± 73	0.003	0.130
$CD3^+$ T cells	7 ± 4	4 ± 2	81 ± 65	58 ± 38	0.120	0.386
$CD19^+$ B cells	2 ± 3	0.6 ± 0.8	8 ± 10	5 ± 7	0.313	0.484
$CD11c^+$ APCs	0.7 ± 0.6	0.5 ± 0.7	8 ± 7	5 ± 3	0.639	0.354
$CD15^+$ PMNs	10 ± 7	0.02 ± 0.02	22 ± 13	0.3 ± 0.3	0.003	0.001
$CD4^+$ T cells	5 ± 3	3 ± 2	58 ± 45	45 ± 30	0.083	0.475
$CD8^+$ T cells	1 ± 0.8	1 ± 1	16 ± 14	9 ± 9	0.362	0.253
$CD4^-$ $CD8^-$ T cells	0.5 ± 0.6	0.5 ± 0.4	3 ± 3	2 ± 2	0.874	0.532
Tregs	0.4 ± 0.3	0.1 ± 0.2	5 ± 4	2 ± 3	0.130	0.173
γδ T cells	0.2 ± 0.4	0.1 ± 0.3	0.3 ± 0.3	0.2 ± 0.2	0.783	0.745
MAIT cells	0.02 ± 0.02	0.02 ± 0.01	0.3 ± 0.3	0.2 ± 0.2	0.632	0.372

[§] p-values from Welch's t test (n = 9). Epithelium (E), Lamina propria (L). °indicates cryopreserved tumors.

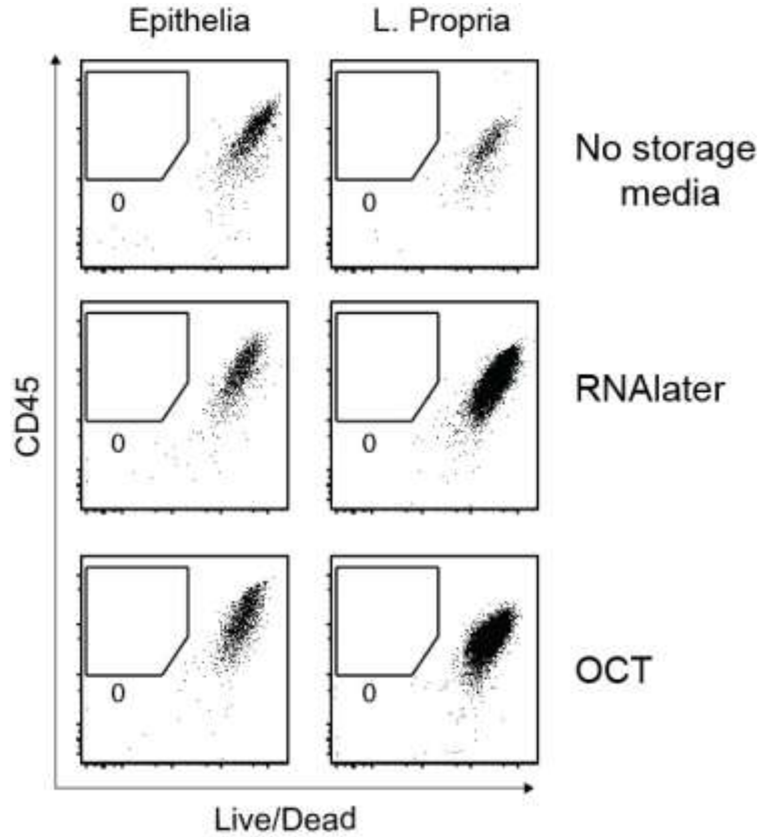

Figure S1. Assessment of TIL recovery with distinct cryopreservation protocols. Representative flow cytometry plots of two experiments showing the absence of viable CD45+ leukocytes in epithelial and lamina propria suspensions of rectal tumors cryopreserved without storage media, or submerged in the frequently used tissue storage media RNAlater and OCT. Percentages of parent shown.

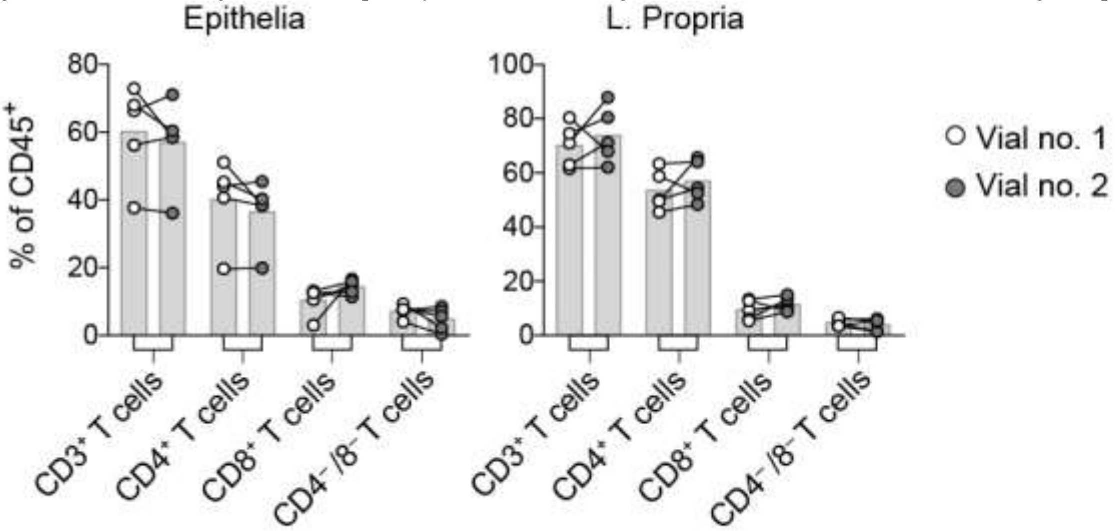

Figure S2. Comparison of TIL frequencies in aliquots of the same rectal tumors. Cryopreserved aliquots (vial 1 and 2 of same tumors) were used at two separate experiment occasions. Frequencies of TIL subsets within live CD45+ leukocytes were compared between these aliquots to assess experimental reproducibility and TIL recovery. Bars show the mean.

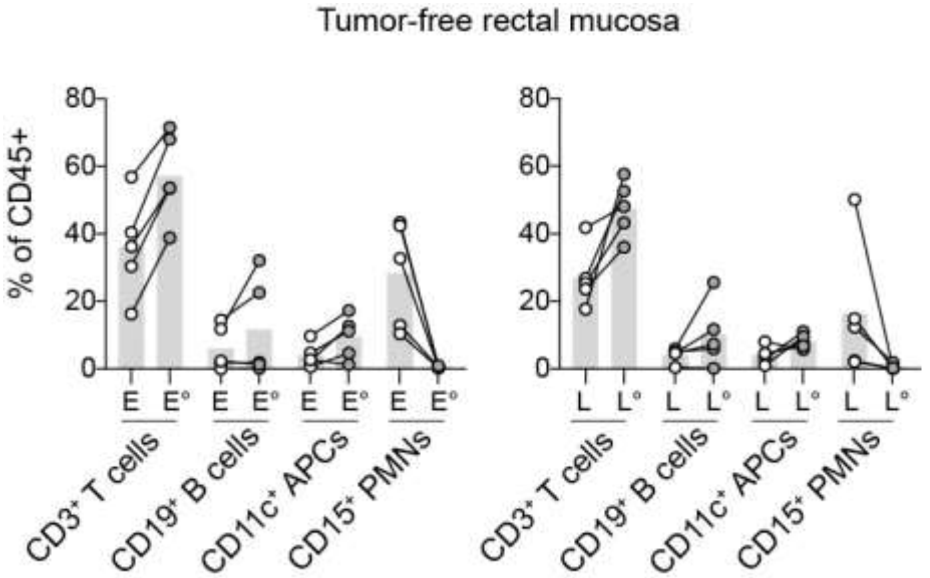

Figure S3. Generation of cell suspensions of cryopreserved rectal mucosa. Pieces of macroscopically tumor-free rectal mucosa were divided in two equal parts. One part was processed into cell suspension immediately and the remaining pieces were cryopreserved and then processed with the same protocol 4-6 weeks later. Percentages of indicated leukocyte subsets within live CD45+ cells were compared in epithelial (E) and lamina propria (L) cell suspensions of fresh vs. cryo-preserved rectal mucosa. Bars show the mean. °indicates the cryopreserved portion.

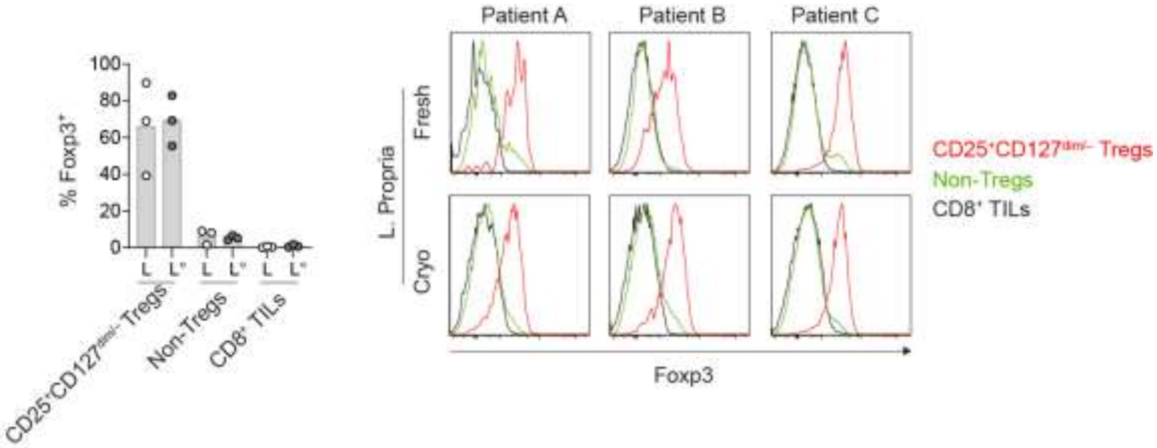

Figure S4. Foxp3 expression by TILs in fresh and cryopreserved rectal tumors. Compiled data on %Foxp3+ population of CD4+CD25+CD127dim/– Tregs, non-Tregs (remaining CD4+ TILs outside the Treg gate) and CD8+ TILs in cell suspensions of lamina propria (L). Bars show the mean. Histograms on Foxp3 staining on indicated TIL subsets in fresh and cryo-preserved portion of the same tumors from three separate patients. °indicates the cryopreserved tumor portion.
